# Supplementary material for: Tonantzin, a New Genus of Bess Beetle (Coleoptera, Passalidae) from a Montane Subtropical Forest in Central Mexico, with a Review of the Taxonomic Significance of the Mesofrontal Structure in Proculini
Source: Insects. 2019 Jun 28;10(7):188. doi: 10.3390/insects10070188 (PMC6681322; doi:10.3390/insects10070188)
Supplement: Supplementary file 1 [file insects-10-00188-s001.zip › Supplementary files/Supplementary table 1.docx]

**Supplementary table S1.** Sequences of primers used for PCR amplification.

| **Gene** | **Primer Name** | **Sequence** | **Source** |
| --- | --- | --- | --- |
| **28s** | rD1.2a | 5’-CCC SSG TAA TTT AAG CAT ATT A-3’ | Whiting 2002 [1] |
| **28s** | rD4.2b | 5’-CCT TGG TCC GTG TTT CAA GAC GG-3’ | Whiting 2002 [1] |
| **28s** | Squirtle | 5’-GTG CAC TTC TCC CCC WGT AG-3’ | Moore et al. 2015 [2] |
| **28s** | rD5b | 5’-CCA CAG CGC CAG TTC TGC TTA C-3’ | Whiting 2002 [1] |
| **CAD** | 439F | 5’- TTC AGT GTA CAR TTY CAY CCH GAR CAY AC-3’ | Wild & Maddison 2008 [3] |
| **CAD** | 668R | 5’- ACG ACT TCA TAY TCN ACY TCY TTC CA-3’ | Wild & Maddison 2008 [3] |
| **CAD** | 688R | 5’- TGT ATA CCT AGA GGA TCD ACR TTY TCC ATR TTR CA-3’ | Wild & Maddison 2008 [3] |

Reference

1. Whiting, M.F. Mecoptera is paraphyletic: multiple genes and phylogeny of Mecoptera and Siphonaptera. *Zool. Scr.* **2002**, *31*, 93–104.

2. Moore, M.R.; Beza‐Beza, C.F.; Wickell, D.A.; Beck, J.B.; Jameson, M.L. Molecules, morphology and Mimeoma scarabs: evolutionary and taxonomic implications for a palm-associated scarab group. *Syst. Entomol.* **2015**, *40*, 891–900.

3. Wild, A.L.; Maddison, D.R. Evaluating nuclear protein-coding genes for phylogenetic utility in beetles. *Mol. Phylogenet. Evol.* **2008**, *48*, 877–891.
